# In-field evaluation of clinoptilolite feeding efficacy on the reduction of milk aflatoxin M1 concentration in dairy cattle

**DOI:** 10.1186/s40781-016-0106-4

**Published:** 2016-06-22

**Authors:** Panagiotis D. Katsoulos, Maria A. Karatzia, Constantinos Boscos, Petra Wolf, Harilaos Karatzias

**Affiliations:** Clinic of Farm Animals, School of Veterinary Sciences, Aristotle University of Thessaloniki, 11 St. Voutyra str, Thessaloniki, 54627 Greece; Institute for Nutrition Physiology and Animal Nutrition, University of Rostock, Justus-von-Liebig-Weg 6b, Rostock, 18059 Germany

**Keywords:** Aflatoxins, Clinoptilolite, Milk, Dairy cows

## Abstract

**Background:**

Clinoptilolite is a natural zeolite with high adsorption capacity for polar mycotoxins such as aflatoxins. The efficacy of clinoptilolite in ameliorating the toxic effects of aflatoxicosis has been proven in monogastric animals, but there is no such evidence for ruminants. The aim of this study was to evaluate, under field conditions, whether the dietary administration of clinoptilolite in dairy cows could reduce the concentration of aflatoxin M1 (AFM_1_) in bulk-tank milk, in farms with higher than or close to 0.05 μg/kg of milk (European maximum allowed residual level). An objective of the present study was also to investigate the effect of particle size of clinoptilolite on aflatoxin binding.

**Methods:**

Fifteen commercial Greek dairy herds with AFM1 concentrations in bulk tank milk ≥0.05 μg/kg were selected. Bulk tank milk AFM1 was determined prior to the onset and on day 7 of the experiment. Clinoptilolite was added in the total mixed rations of all farms at the rate of 200 g per animal per day, throughout this period. Two different particle sizes of clinoptilolite were used; less than 0.15 mm in 9 farms (LC group) and less than 0.8 mm in 6 farms (HC group).

**Results:**

Clinoptilolite administration significantly reduced AFM_1_ concentrations in milk in all farms tested at an average rate of 56.2 % (SD: 15.11). The mean milk AFM_1_ concentration recorded on Day 7 was significantly (*P* < 0.001) lower compared to that of Day 0 (0.036 ± 0.0061 vs. 0.078 ± 0.0074 μg/kg). In LC group farms the reduction of milk AFM_1_ concentration was significantly higher than HC group farms (0.046 ± 0.0074 vs. 0.036 ± 0.0061 μg/kg, *P* = 0.002). As indicated by the Pearson correlation, there was a significant and strong linear correlation among the milk AFM_1_ concentrations on Days 0 and 7 (R = 0.95, *P* < 0.001).

**Conclusions:**

Dietary administration of clinoptilolite, especially of smallest particle size, at the rate of 200 g per cow per day can effectively reduce milk AFM_1_ concentration in dairy cattle and can be used as a preventive measure for the amelioration of the risks associated with the presence of aflatoxins in the milk of dairy cows.

## Background

Aflatoxins (AF) are hepatotoxic and carcinogenic secondary metabolic products from fungi belonging in particular to the *Aspergillus flavus* and *A. parasiticus* species [1, 2]. Aflatoxin B1 (AFB_1_) is of major concern in dairy cattle. When fed to lactating animals, a part of this AF is destroyed in the rumen, whereas the absorbed quantity is rapidly oxidized mainly to aflatoxin M1 (AFM_1_) [3–5]. Most of the AFM_1_ produced is excreted in the urine and less so in the milk [6, 7]. The carry-over of AFB_1_ to AFM_1_ in milk depends on many factors such as milk yield and days in lactation [8–11] and ranges between 1 % and 6 % [11, 12]. As AFM_1_ is a possible human carcinogen [13], many countries have applied a maximum residue level for this aflatoxin in ruminant milk. For the EU this level is 0.05 μg/kg [12].

In dairy farms milk AFM_1_ concentration often either approaches or exceeds the aforementioned maximum residue level. In these cases it is common practice to add a mycotoxin binder in the ration until the detection and withdrawal of the contaminated feed. The role of these binders is to adsorb and reduce the intestinal absorption of mycotoxins to reduce the toxic effects for livestock and the carry-over of toxin compounds to animal products. Clinoptilolite is a natural zeolite, licensed by the EU as additive in feedstuffs of farm animals [14]. This material has high adsorption capacity for polar mycotoxins such as aflatoxins [15–17]. Concerning AFB_1_ specifically, it has been determined that 1 g of clinoptilolite can adsorb about 200 μg of this aflatoxin [16]. The efficacy of clinoptilolite in ameliorating the toxic effects of aflatoxicosis has been proven in monogastric animals, especially in poultry [18–23]. Such trials scarce from the available literature for ruminants and there is no reference providing information about the potential use of clinoptilolite as aflatoxin binder in dairy cattle. However, the use of other alumonosilicate binders closely related to clinoptilolite such as hydrated sodium calcium aluminosilicate was proven to be effective in reducing AFM_1_ in milk of cattle [24] and goats [25] in experimentally induced aflatoxicosis.

Based on this evidence, the present study was designed in order to evaluate, under field conditions, whether the dietary administration of clinoptilolite in dairy cows could reduce the concentration of aflatoxin M1 (AFM_1_) in bulk-tank milk, in farms with higher than or close to 0.05 μg/kg of milk (European maximum allowed residual level). An objective of the present study was also to investigate the effect of particle size on the mycotoxins binding capacity of clinoptilolite.

## Methods

The study was conducted in 15 commercial Greek herds with Holstein dairy cattle that had high concentrations of AFM_1_ in bulk tank milk (close to or higher than 0.05 μg AFM_1_/kg of milk), as detected at the routine analyses by the dairy industries collecting the milk. Milk AFM_1_concentration was determined using their standard methods (ELISA) following the guidelines and recommendations described in ISO 14675/2003. The criteria for the selection of the farms were: a) the willingness of the farmers to use only clinoptilolite as mycotoxin binder without making any other changes to the rations offered to the animals during the experimental period b) the agreement of the milk industries to determine the AFM_1_ concentrations in the delivered bulk tank milk at fixed time-points and c) the difference between bulk tank milk AFM_1_ concentration at the initial measurement and at the onset of the experiment (3–10 days later) not being more than 10 %. In all these farms the total mixed rations offered to the animals were formulated by the attending animal scientists and met the requirements of the cows for maintenance and respective milk production. Herd size, average milk production per cow per day, average daily dry matter intake and farming system are presented in Table 1.

**Table 1:** Herd size, farming system (Free stall: FS; Cubicle with earthen paddocks: CEP), average daily milk yield per cow, dry matter intake (DMI), contaminated feeds in the ration, inclusion rate of clinoptilolite (% dry matter; % DM) and grouping according to the particle size of clinoptilolite (SC: <0.15 mm, LC: <0.8 mm) in the 15 farms (F1 to F15) included in the study

Group	Farm I.D.	Herd Size	Farming system	Average daily milk yield (kg)	DMI (kg)	Clinoptilolite inclusion rate (% DM)	Contaminated feed^a^
SC	F1	220	CEP	32	20.6	0.97	Corn silage
SC	F2	246	CEP	30	20	1.00	Corn silage
SC	F5	125	CEP	30	20	1.00	DDGS^b^
SC	F6	172	FS	33	20.5	0.98	Maize grains & cottonseed meal
SC	F8	50	FS	25	18.8	1.06	Maize grains
SC	F9	87	FS	27	19.2	1.04	Maize grains
SC	F10	120	CEP	31	20.2	0.99	Maize grains
SC	F11	136	CEP	36	22.0	0.91	Maize grains
SC	F13	175	FS	29	19.9	1.01	N/D^c^
LC	F3	180	FS	28	19.5	1.03	DDGS^b^
LC	F4	146	FS	33	20.8	0.96	Corn silage
LC	F7	163	CEP	29	19.7	1.02	Maize grains
LC	F12	90	FS	35	21.8	0.92	Maize grains
LC	F14	74	FS	26	18.7	1.07	N/D^c^
LC	F15	150	FS	33	20.2	0.99	N/D^c^

^a^According to farmers’ statement

^b^Distiller’s dried grains with solubles

^c^Not determined

The study lasted for 7 days. On Day 0, the bulk tank milk AFM_1_ concentration was determined. For the next seven days (Days 1 to 7), clinoptilolite was added in the total mixed rations of all farms at the level of 200 g per animal per day. The rate of clinoptilolite supplementation (% of the dry matter of the ration) is shown in Table 1. The bulk tank milk AFM_1_ was also determined at the delivered milk produced on Day 7 of the experiment. The aflatoxin contaminated feedstuffs in the rations of 12 out of 15 farms, as stated by the farmers that analyzed their feedstuffs in private laboratories on their own initiative are presented in Table 1.

### Clinoptilolite

The clinoptilolite-rich zeolitic material (Vivolith 85, S&B, Greece) used in all farms contained at least 85 % (85 %–87 %) clinoptilolite according to the analyses provided by the manufacturer for the batches used; the admixtures were feldspar, micas and clays as determined by x-ray powder diffraction. The cation exchange capacity of the material used was 160 mEq/100 g and its chemical composition was as follows: SiO_2_ 68.3 %, Al_2_O_3_ 12.8 %, Fe_2_O_3_ 1.1 %, CaO 3.2 %, MgO 0.7 %, K_2_O 3.4 %, Na_2_O 1.0 % and loss on ignition (LOI) 8.5 %.

According to the size of zeolite, farms were allocated into two groups. The first group (SC) consisted of 9 farms that used a particle size of less than 0.15 mm and the second group (LC) of the other 6 farms that used a particle size of less than 0.8 mm (Table 1). The allocation to these groups was completely randomized.

### Ethics

The procedures and the experiment were done according to the ethical standards in the Helsinki Declaration of 1975, as revised in 2000, as well as the national law and the guidelines of our Institutional Animal Care and Use Committee.

### Statistical analysis

Data were analyzed using the statistical program SPSS® 21. Normality of data distribution was assessed with Kolmogorov-Smirnov test and homogeneity of variances was evaluated with Levene’s test. Repeated measures analysis was run to evaluate the significance of the differences among the AFM1 concentrations in the bulk tank milk before (Day 0) and after the administration of clinoptilolite (Day 7). Univariate analysis of variance was also used for the comparison of the absolute differences (AD) and the absolute relative differences (ARD) of the AFM_1_ concentration in milk at the onset and the end of the study period between the two groups (SC and LC) of farms (AD = AFM_1o_- AFM_1F_ and ARD = 100xAD/AFM_1o_; where AFM_1F_ represents the milk AFM_1_ concentration on Day 7 and AFM_1o_ the milk AFM_1_ concentration on Day 0). Bonferroni test was used as an adjustment factor for the comparison of the main effects of time and group respectively in the former models. The linear association between the milk AFM_1_ concentrations on Days 0 and 7 was assessed by the use of the Pearson Correlation Coefficient test and Linear Regression Analysis was used to determine the equations predicting the milk AFM_1_ concentration after clinoptilolite administration. A value of *p* ≤ 0.05 was considered significant in all comparisons. The sensitivity and the specificity for the correct prediction of individual farms with or without milk AFM_1_ concentration over 0.05 μg/kg after clinoptilolite administration using the generated equation was calculated using MedCalc Diagnostic test evaluation calculator (available at: https://www.medcalc.org/calc/diagnostic_test.php).

## Results

The inclusion rate of clinoptilolite in the total mixed rations of the 15 farms included in the study is presented in Table 1. On average, the inclusion rate was 1 % (SD: 0.05) of the ration’s dry matter (DM).

**Fig. 1:**
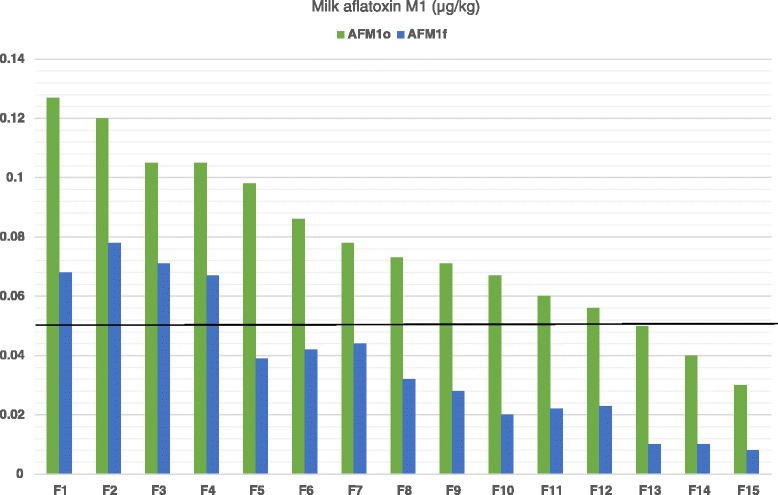
Milk aflatoxin M1 concentrations (μg/kg) on days 0 (AFM1o) and 7 (AFM1f) of the experiment in the 15 farms (F1 to F15) included in the study

**Fig. 2:**
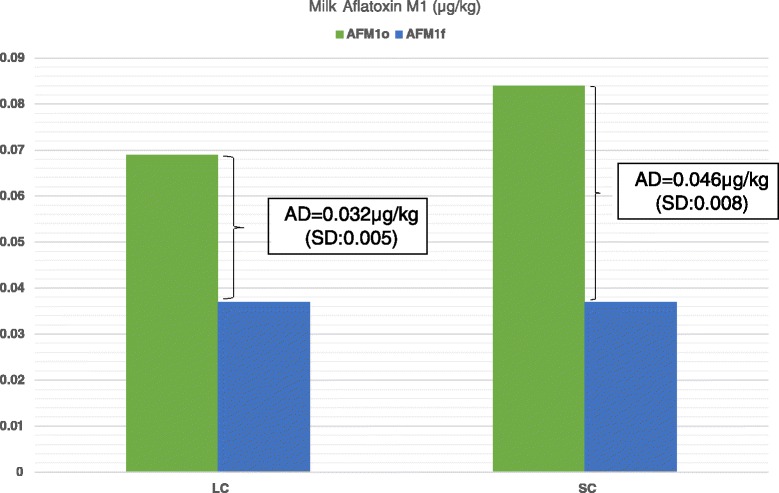
Milk aflatoxin M1 concentrations (μg/kg) on days 0 (AFM1o) and 7 (AFM1f) of the experiment in the 6 farms of LC group (particle size of clinoptilolite <0.8 mm) and in the 9 farms of SC group (particle size of clinoptilolite <0.15 mm) and the absolute differences (AD) recorder in each group

As it is shown in Fig. 1, reduced milk AFM_1_ concentrations were recorded in all farms tested after the dietary inclusion of clinoptilolite (Day 7) compared to Day 0. On average, milk AFM_1_concentration on Day 7 was significantly lower compared to Day 0 (mean ± SE: 0.078 ± 0.0074 μg/kg and 0.036 ± 0.0061 μg/kg for Days 0 and 7, respectively, *P* < 0.001). Moreover, the average AD was 0.04 μg/kg (SD: 0.01) and the average ARD was 56.2 % (SD: 15.11). In farms of the LC group the average AD of milk AFM_1_ concentration was significantly higher than that in farms of HC group (mean ± SE: 0.046 ± 0.0074 μg/kg and 0.036 ± 0.0061 μg/kg for groups LC and HC, respectively, *P* = 0.002; Fig. 2). However, the average ARD was not significantly different among groups (mean ± SE: 58.1 ± 13.15 % and 53.2 ± 18.6 % for groups LC and HC, respectively, *P* = 0.56).

**Fig. 3:**
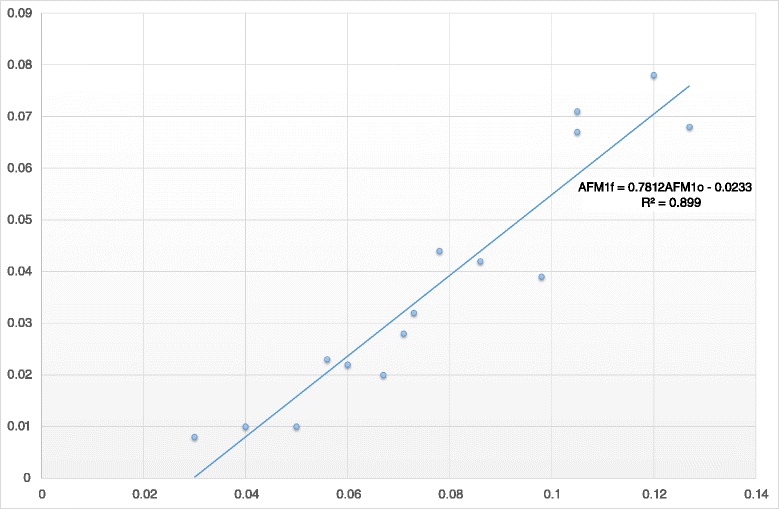
Scatterplot with linear regression equation of the milk aflatoxin M1 concentration on days 0 (AFM1o) and 7 (AFM1f) of the experiment

As indicated by the Pearson correlation, there was a significant and strong linear correlation among the milk AFM_1_ concentrations on Days 0 and 7 (R = 0.95, *P* < 0.001). According to the results of regression analysis, the equation yielding the predicting value of milk AFM_1_ concentration after the administration of clinoptilolite (Day 7) was:

$$ \mathsf{A}\mathsf{F}{\mathsf{M}}_{\mathsf{1F}} = \mathsf{0}.\mathsf{781}\times \mathsf{A}\mathsf{F}{\mathsf{M}}_{\mathsf{1o}}\hbox{--}\ \mathsf{0}.\mathsf{023},\ \left({\mathsf{r}}^{\mathsf{2}}=\mathsf{0.899},\ \mathsf{P}<\mathsf{0.001}\right), $$

where AFM_1F_ represents the milk AFM_1_ concentration on Day 7 and AFM_1o_ the milk AFM_1_ concentration on Day 0 (Fig. 3).

The sensitivity of the correct prediction of individual farms with or without milk AFM_1_ concentration over 0.05 μg AFM_1_/kg after clinoptilolite administration using this equation was 100 % and the specificity was 90.91 %.

## Discussion

The objective of the present study was to evaluate the efficacy of a natural zeolite; clinoptilolite, as aflatoxin binder in dairy cattle. It was selected to run the study in natural cases rather than experimentally induced aflatoxicoses which is the common practice in most of the studies [9, 26, 27] evaluating other mycotoxin binders. It was done not only for welfare reasons but also because such a study design would allow us to test the efficacy of clinoptilolite under field conditions and to have a considerable number of replications by using different farms. Besides, the in vitro and the in vivo studies that preceded in other animal species [15–23] provided enough evidence for potential efficiency of the binder on the reduction of aflatoxins. The criteria of farm selection were set in order to ensure, as most as possible, that the milk AFM_1_ concentration on day 0 represents a stable situation of high milk AFM_1_ and that the reductions detected on day 7 are due to the in-feed inclusion of clinoptilolite. The dosage rate of clinoptilolite offered daily to the animals was chosen to be 200 g per animal per day because this amount is safe for long-term consumption and has beneficial effects on the health status and the performance of dairy cows [28]. Furthermore, this daily dose represented the 1 % of ration DM, on average, which is within the rates most commonly used for the evaluation of a mycotoxin adsorbent in dairy cows [9, 26, 27]. According to our observations during a series of experiments [28], the addition of clinoptilolite at this level in the total mixed rations has no effect on the feed consumption even from the first day of administration. So, no gradual increase of the amount of clinoptilolite fed was judged to be necessary prior to the onset of the evaluation period and the selected daily dose was offered to the animals from the first day of the study. The duration of 7 days was considered to be adequate as study period given that it is about double the clearance time after the removal of aflatoxins of the dairy cattle diet which is about 3–4 days [9, 10, 27].

The results obtained here prove that the dietary administration of clinoptilolite is effective in reducing the milk concentration of AFM_1_, given the practically stable AFM_1_ milk concentration from the first detection by the milk industries until the onset of the experiment and the continuous use of the same rations without any change until the end of the experiment. With the exception of the 4 farms where the initial milk AFM_1_ concentration was higher than 0.1 μg/kg, in all other cases the final AFM_1_ concentration was below the maximum residue level of 0.05 μg/kg. This indicates that the dosage rate of 200 g clinoptilolite per cow per day, is adequate for the prevention of aflatoxicosis with AFM_1_ milk concentrations lower than 0.1 μg/kg. The average relative reduction of milk AFM_1_ recorded at this study, although not comparable with other experiments due to different study design, was similar to those achieved with other aluminocilicate mycotoxin binders; Diaz et al. [9] observed that calcium and sodium bentonite products reduce milk AFM_1_ concentrations by 31 % to 65 % and Kutz et al. [25] that two hydrated sodium calcium aluminocilicates decrease the AFM_1_ by 45 and 48 %. The relatively high standard deviation, about 25 % of the means, of the AD and the ARD recorded is probably due to the wide range of initial milk AFM_1_ concentrations, to the large variation of the carry-over rate of AFB1 to AFM1 in milk [8–11] and to the differences of the rations offered to the animals among farms.

The use of clinoptilolite-rich material with the particle size of less than 0.15 mm resulted in significantly higher decrease of milk AFM_1_ concentration than the one of less than 0.8 mm. Due to the porous nature of clinoptilolite, the smaller particle size provides a larger surface that is available for interaction with the polar mycotoxins such as aflatoxins [29]. So, the <0.15 mm clinoptilolite adsorbed higher quantities of AFB_1_ in the gastrointestinal tract than the <0.8 mm one. The absence of significant difference on the average absolute relative reduction between groups is associated with the higher average milk AFM_1_ concentrations recorded on the LC group compared to HC.

Another interesting finding was the strong linear relationship among the initial and the final AFM_1_ milk concentration and the high value of the coefficient of determination of the regression equation produced. The very high sensitivity and specificity values determined denote that using this equation it is possible to predict whether the milk AFM1 concentration will be lower than the maximum residue level of 0.05 μg/kg or not. If not, the simultaneous use of another aflatoxin binder or the administration of higher amounts of clinoptilolite should be considered given that the adsorption of aflatoxins by aluminosilicate binders occurs in a dose dependent manner [30]. Besides, higher daily doses of clinoptilolite than 200 g in dairy cattle are safe even for long-term administration [28].

## Conclusions

In the context of this study it can be concluded that the dietary administration of clinoptilolite, especially of smallest particle size, can effectively reduce milk AFM_1_ concentration in dairy cattle. Taking into consideration the relatively low commercial price of clinoptilolite (0.30-0.40 Euros per kg) and that the long-term dietary supplementation of clinoptilolite is safe and has beneficial effects on the health status and productivity of dairy cattle, without adding any extra labour in the farm’s routine, clinoptilolite feeding at the rate of 200 g per cow per day can be used as a preventive measure for the amelioration of the risks associated with the presence of aflatoxins in the milk of dairy cows. Of course, further research using higher inclusion rates of clinoptilolite is regarded necessary in order to evaluate its efficacy when bulk tank milk AFM_1_ levels are higher than 0.1 μg/kg.

## Abbreviations

AFM1, aflatoxin M1; AFB1, aflatoxin B1; LC, clinoptilolite with <0.15 mm particle size; HC, clinoptilolite with <0.8 mm particle size; SD, standard deviation; SE, standard error; AFM1F, milk aflatoxin M1concentration on Day 7; AFM1o, milk aflatoxin M1 concentration on Day 0; AD, absolute differences; ARD, absolute relative differences; DM, dry matter; DMI, dry matter intake; FS, free stall; CEP, Cubicle with earthen paddocks.
